# Depth Without a Surface: Observations From a “Finger Spinner” Depth Illusion

**DOI:** 10.1177/2041669517740370

**Published:** 2017-11-16

**Authors:** Michael A. Crognale

**Affiliations:** University of Nevada, Reno, NV, USA

**Keywords:** 3D perception, surfaces/materials, stereopsis, binocular vision, depth

## Abstract

A trending novelty toy that is spun between the fingers induces a striking depth illusion from specular reflections. Further examination of the phenomenon suggests that when surface features are obscured by spinning, depth from disparity of reflections is enhanced.

“Finger spinners” or “Fidget spinners” are a trending toy comprising weighted shapes with a central bearing which can be held by the fingers and spun rapidly, apparently for a modicum of amusement. I received a rather cheap, but shiny, chrome version of one of these (see Figure 1) as a novelty gift and dutifully spun it to reap my promised joy. I was not disappointed. Spinning the toy produced the appearance of eccentric, luminous rings created by specular highlights (see Figure 2). These highlights were the reflections of numerous room lights. This was not particularly surprising. However, each ring appeared at varying depths that greatly exceeded the thickness of the spinner. In fact, my thumb, which was placed on the top, appeared to be imbedded in a cone created by one of the rings. In addition, there was ambiguity in the percept such that two overlapping rings at different depths could appear as crescent moons, rather than as two overlapping rings. Surprisingly, when the percept was the moon shape, the depth differences of the original rings were maintained such that the concave portion of the moon appeared to be at a different depth than the convex portion. Consequently, the shape appeared to bend in three dimensions. Importantly, the depth vanished when viewed monocularly, even though the ambiguous ring/moon shapes were still visible, implicating disparity as a likely source of the effect. The depth effect was equally compelling with a single-point source of light such as the sun albeit with fewer visible rings.

**Figure 1.**
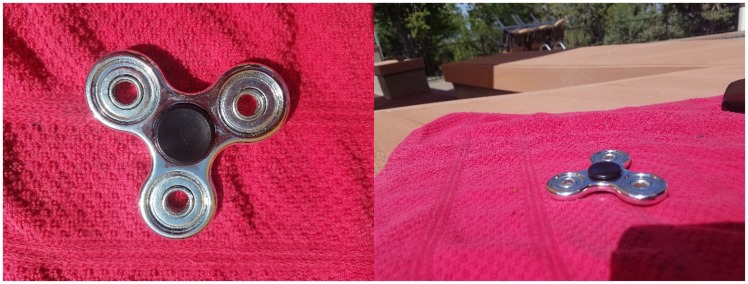
Finger spinner viewed from above and from an oblique angle to illustrate specular highlights.

**Figure 2.**
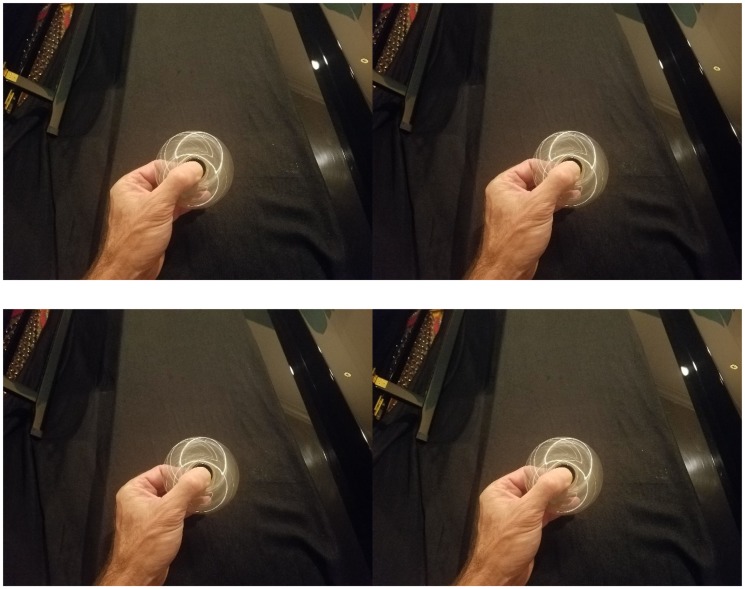
Stereoscopic images of the depth effect. Converging fusers use the top pair. Diverging fusers use the bottom pair.

In an informal polling, 100% of 25 adult observers confirmed both the depth and the bi-stable “moon” effects as well as the binocularity requirement (although two observers reported some lingering depth under monocular conditions). Also, most observers reported no depth (reflection bound to the surface) when the spinner was static. Some observers (including a reviewer) report seeing less but some depth in the static condition.

Given the requirement for binocular disparity, a geometric explanation for the illusion may suffice. If one examines the shape of the spinner, it is clear that in the horizontal plane there are areas of convexity and areas of concavity (since the surface is toroidal and not spherical, there is convexity but no concavity in the orthogonal plane). For example, the outside surfaces of the areas surrounding the three toroidal regions at the apexes of the spinners are convex. Conversely, the areas in between the toroidal regions have negative or concave curvature (in the horizontal plane). A specular reflection from a distant source viewed in a convex surface will shift concurrently with a shift in viewing angle in the same direction. Consequently, the reflection viewed from the right eye will be further to the right on the surface than when viewed from the left eye. This geometric arrangement is known as uncrossed disparity. As is well established, uncrossed disparity as a cue for stereopsis results in a percept of depth beyond the horopter (perceived as a relatively greater distance). Conversely, the concave surface causes the opposite shift of the reflection; it is further to the left on the surface when viewed from the right eye than when viewed from the left eye. This geometry known as crossed disparity results in a percept of a closer object. Thus, the shape of the spinner and the geometry of the optics predict the anecdotal observations reported earlier.

Although the aforementioned geometric explanation threatens to trivialize the significance of the illusion, it is important to consider why such depth effects are less obvious when the spinner is static or in other instances of specular reflections on curved surfaces in everyday life. Often, disparity between fused images results in either a percept of depth or in binocular rivalry. In the case of a specular reflection on a static curved surface, there is often neither. Rather, as shown previously (e.g., Blake & Bülthoff, 1990; Sakano & Ando, 2010), disparity can be used to infer surface glossiness and curvature but is often ignored as a cue to source distance. In the static condition of the spinner, there are surface features such as contours and diffuse reflection that unambiguously define the three-dimensional structure of the spinner in terms of stereoscopic and other depth cues. Such cues may provide enough information to partially discount the disparity cues from the specular reflections. However, when the spinner is rotating, these surface features are obscured, leaving only the trail of the moving reflections with disparity and consequent depth; that is, depth without a surface.

One prediction from the explanation posited above is that specular reflections from other concave and convex surface placed in motion to obscure the local surface properties should produce similar depth effects. Since I apparently have a knack for breaking sunglasses, I tested this by attaching two cheap sunglass lenses to a stick with opposite orientations of the convex surface (see Figure 3). Rotating the stick in the presence of a point source of light produced a strong percept of depth consistent with the hypothesis, that is, reflections from the convex lens appeared much further away than reflections from the concave lens. In fact, the appearance of depth was strong enough that the lower arc appeared to pass right through the hand that was holding the device.

**Figure 3.**
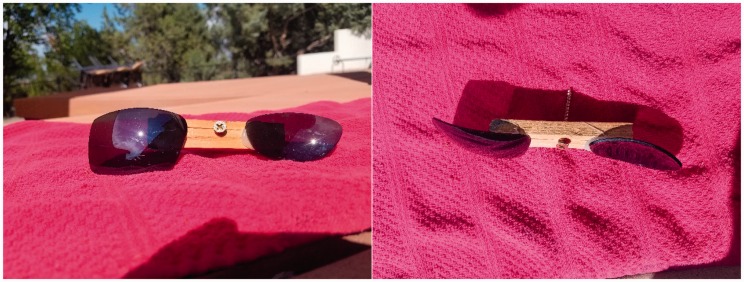
Sunglass lenses attached to a short stick in opposite orientations.

One further note is that when the reflections are images of objects rather than point sources, the static concave surface can induce strong depth from disparity, making images of objects appear to float, as has been noted previously in the literature. For example, an image of an extended fluorescent light fixture appears to float above the concave lens of Figure 3. It appears that increased information available in the image of objects reflected from convex surfaces is not as easily discounted.

The illusion raises number of interesting questions: (a) How much surface information is required to null the depth of the reflection and anchor it to the surface? (b) Is the discounting of disparity in the image an automatic process or does it have to be learned through experience (like the bumps and divots illusion? (c) If discounting is learned, what is the time course of development (e.g., over a similar time course as that of discounting the illuminant in color constancy)?

In any case, I am reluctantly forced to admit that the trending toys known as “finger spinners” may indeed be sources of pleasure, or at least contemplation.
